# Modification of the Low FODMAP Diet Is Feasible in the Treatment of Irritable Bowel Syndrome: A Randomised Crossover Study

**DOI:** 10.1155/grp/4152978

**Published:** 2025-11-13

**Authors:** Line Graser Jensen, Marie Kjær, Jens Rikardt Andersen

**Affiliations:** Department of Nutrition, Exercise and Sports, Faculty of Science, University of Copenhagen, Copenhagen, Denmark

**Keywords:** fructooligosaccharides, fructose, galactooligosaccharides, irritable bowel syndrome, low FODMAP diet, polyols

## Abstract

**Background:**

Irritable bowel syndrome (IBS) can be dietary managed by applying restrictions in the diet of fermentable oligosaccharides, disaccharides, monosaccharides and polyols—the low FODMAP diet. However, many patients have major difficulties integrating the diet into their daily lives.

**Objective:**

We aimed to investigate if the three carbohydrate groups eliminated in the traditional low FODMAP diet are equally important in relieving gastrointestinal symptoms in IBS.

**Methods:**

Nine patients with IBS according to the Rome IV criteria and referred to specialised diet therapy in private clinics were randomised in a crossover design to three different carbohydrate-modified diets: (A) low polyol diet, (B) low FOS + GOS diet and (C) low standard FODMAP diet for 4 weeks on each diet. Symptoms were assessed by the Birmingham IBS questionnaire and adequate relief (IBS-AR) and quality of life by the IBS Quality of Life Scale questionnaire (IBS-QOL) at baseline and after every intervention period by a dietitian with assessment of the intake by weekly contact. Nonparametric statistical methods were used.

**Results:**

Compared to baseline, the low polyol diet did not change the symptoms, but relief was significant on both the low FOS + GOS diet and the low FODMAP diet (*p* < 0.05) with no difference between these two diets. Clinically relevant symptom relief was experienced by 75% of patients on the low FOS + GOS diet and 62.5% on the low FODMAP diet, but none on the low polyol diet.

**Conclusion:**

A carbohydrate-modified diet with the exclusion of fructooligosaccharides and galactooligosaccharides (low FOS + GOS diet) reduced gastrointestinal symptoms and improved quality of life equally to the standard low FODMAP diet in patients with IBS. Polyol restriction was of minor importance. The low FOS + GOS diet could be the starting diet in selected patients with IBS.

**Trial Registration:**

ClinicalTrials.gov identifier: NCT05618106

## 1. Introduction

Irritable bowel syndrome (IBS) is the most common functional gastrointestinal disorder affecting 10%–20% of the population globally and is characterised by chronic symptoms such as recurrent abdominal pain in combination with bloating as well as diarrhoea, constipation or both [1, 2]. The symptoms are often severe, and IBS can substantially impact patients' health-related quality of life and is associated with increased healthcare utilisation and has socioeconomic consequences [2–6]. However, even though IBS is a highly prevalent gastrointestinal disorder, the pathophysiology is poorly understood [4].

Dietary restriction of highly fermentable oligosaccharides, disaccharides, monosaccharides and polyols (low FODMAP diet) is a well-documented therapeutic measure in the symptomatic management of IBS [7–9]. The underlying hypothesis of the low FODMAP diet suggests that restricting the intake of these groups of poorly absorbed and highly fermentable short-chain carbohydrates reduces intestinal osmolarity and gas production, thus reducing gastrointestinal symptoms, particularly pain, bloating and diarrhoea, thus improving the quality of life [10, 11]. The low FODMAP diet is considered the first-line treatment in patients with IBS, with a clinical response in 50%–76% of patients [12–15]. However, patients find it difficult to comply with the low FODMAP diet due to the extensive elimination of foods in their daily life [16, 17]. There is increasing evidence that the standard low FODMAP diet can be simplified to increase compliance [9, 18–20].

Due to the highly restrictive nature of the standard low FODMAP diet, we aimed to investigate if all carbohydrate groups eliminated in the low FODMAP diet are equally important in relieving gastrointestinal symptoms in IBS.

## 2. Material and Methods

### 2.1. Study Participants

Consecutive (recruited in the order of appearance in the clinics without preselection besides the exclusion criteria mentioned below) patients, age 18–65 years and with a BMI of 18.5–25.0 kg/m^2^ (to avoid the influence of weight-reducing diets), diagnosed with IBS according to the Rome IV criteria [17] were recruited from private outpatient clinics specialised in gastroenterology in the Copenhagen area after exclusion of other diseases responsible for their symptoms.

All patients were referred for specialised dietary treatment and had to pay for this in the normally free Danish health system, thereby being highly motivated. All patients were of Subtype M. Patients were not included if they had problems with the Danish language, had structural gastrointestinal diseases, were pregnant, eliminated foods from their habitual diet, were medicated with drugs that could potentially interfere with the end points or had a prior history of eating disorders. None of the patients received medications for chronic diseases.

### 2.2. Study Design and Randomisation

**Figure 1:**
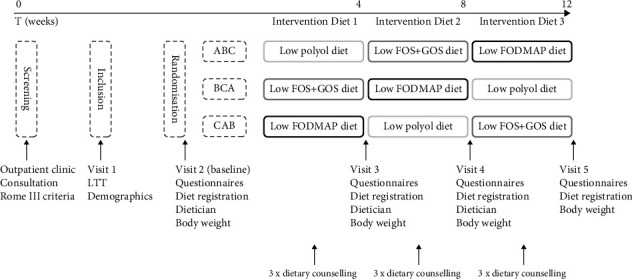
Overview of the study protocol for patients with IBS testing three variations of the low FODMAP diet in a crossover design. The dotted line indicates the randomisation sequence determining the order of the intervention diets: ABC, BCA or CAB. A: low polyol diet, B: low FOS + GOS diet, C: low FODMAP diet. T, time; LTT, lactose tolerance test; FOS, fructooligosaccharides; GOS, galactooligosaccharides.

The study design, timeline and measurements are illustrated in Figure 1. Patients were randomised in a crossover design to three intervention diets for 4 weeks each without wash-out periods for a total period of 12 weeks. Patients were allocated by sealed opaque envelopes with three different sequences determining the order of the intervention diets, ABC, BCA or CAB, as standard block randomisation.

### 2.3. Intervention

The intervention consisted of three different carbohydrate-modified diets in randomised order: (A) low polyol diet, (B) low fructooligosaccharides (FOS) + galactooligosaccharides (GOS) diet and (C) low FODMAP diet. The three intervention diets were based on the original low FODMAP principles [8], where foods included or excluded were based on data from the Monash University database regarding the content of FODMAPs in foods [21] The low polyol diet (A) excluded foods containing polyols. The low FOS + GOS diet (B) excluded oligosaccharides (FOS and GOS). The low FODMAP diet (C) excluded oligosaccharides, disaccharides, monosaccharides and polyols. For all three intervention diets, free fructose in the range of a maximum of three pieces of fruit per day was allowed free of choice to improve compliance with the study protocol. Traditional IBS dietary advice was given routinely and kept constant during the three treatment periods along with the special instructions.

Prior to every intervention diet, all patients were instructed by an experienced, certified clinical dietitian on how to adhere to each specific diet. In addition, a list of foods to be included or excluded for each diet, together with daily meal suggestions, was provided and reviewed with the patients. During every intervention period, the patients received weekly dietary counselling, either as outpatient visits or phone calls by the same dietician. Diet registrations were performed to ensure adherence to the intervention diets for all patients.

### 2.4. Outcomes

The primary outcome of the study was the change in gastrointestinal symptoms assessed by the Birmingham IBS questionnaire [22] from baseline and after each intervention diet. Secondary outcomes were the proportion of patients who experienced adequate relief of their IBS symptoms (IBS-AR) and the change in health-related quality of life (IBS-QOL). All questionnaires were translated into Danish.

### 2.5. Questionnaires

The questionnaires at baseline and after the completion of each intervention diet during outpatient visits included the following: Birmingham IBS symptom questionnaire [22], adequate relief of IBS symptoms (IBS-AR) [23] and IBS Quality of Life Scale questionnaire (IBS-QOL) [24].

### 2.6. Assessment of IBS Symptom Severity

Gastrointestinal symptoms were assessed by the Birmingham IBS, a disease-specific tool developed to measure the presence and severity of IBS symptoms. The Birmingham IBS symptom questionnaire consists of 11 questions based on the frequency of IBS symptoms on a 6-point Likert response scale, ranging from 0 (*none of the time*) to 5 (*all the time*). Responses are presented as a total score and scores for each of the three underlying variables: pain, diarrhoea and constipation [19]. Higher scores indicate higher symptom severity. The adequate relief of IBS symptoms assessment (IBS-AR), a dichotomous response question, was applied to assess whether the patients had experienced *adequate or satisfactory relief of their IBS symptoms* because of the intervention diets.

### 2.7. Assessment of Quality of Life

Health-related quality of life was evaluated by the IBS-QOL questionnaire [24]. The IBS-QOL questionnaire is a validated disease-specific tool used to assess the impact of gastrointestinal symptoms and the effects of treatment for IBS and is composed of 34 questions evaluating eight subscale domains found to be of relevance for patients with IBS: dysphoria, interference with activity, body image, health worry, food avoidance, social reaction, sexual and relationships. Each item has a 5-point Likert response scale, ranging from 0 (*not at all*) to 5 (*extremely*). In the present study, the individual responses to the IBS-QOL questionnaire were summed and averaged for a total score, with lower scores indicating improved quality of life [24].

### 2.8. Statistical Analysis

The Wilcoxon paired-rank test and Friedman rank-sum test were applied to assess changes in Birmingham IBS symptom scores and IBS-QOL scores. Dichotomous variables for patient-reported adequate relief of IBS symptoms were compared using Fisher's exact test. Categorical variables were presented as numbers or percentages, and continuous variables were presented as the mean ± SD. The intention to treat population was defined as patients who were assessed more than one time since baseline. The level of statistical significance was set to *p* < 0.05. The sample size calculation (*n* = 9) was based on 80% power, a level of significance of 0.05 (two-sided) and a need for a difference in symptoms of 20% to be clinically relevant.

### 2.9. Ethical Statements

The study was approved by the Committees of Health Research Ethics in the Capital Region of Denmark (H62014044) and the Danish Data Protection Agency (2012-58-0004). Oral and written informed consents were obtained from all study participants, and the study was in accordance with the Principles of Helsinki.

## 3. Results

**Figure 2:**
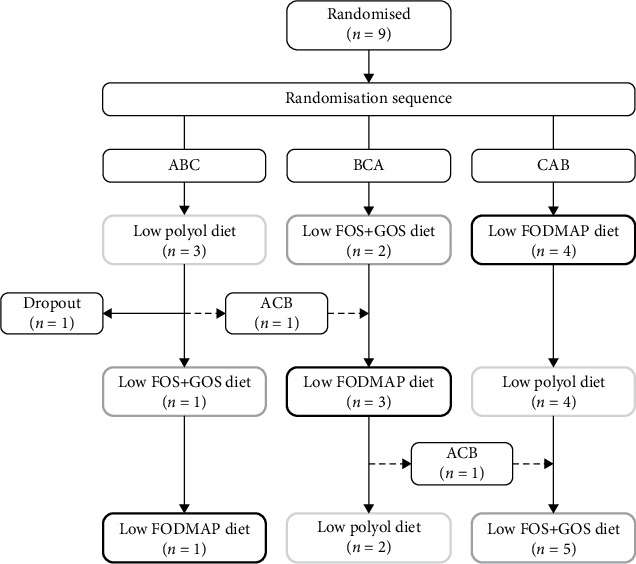
Participant flowchart. Nine patients were included in the study, and one patient dropped out after the first intervention period due to bad tolerance of the low polyol diet. One patient changed randomisation sequence from ABC to BCA. A: low polyol diet, B: low FOS + GOS diet, C: low FODMAP diet. FOS, fructooligosaccharides; GOS, galactooligosaccharides.

The patient's demographics are presented in Table 1. Nine patients were randomly allocated to a randomisation sequence ABC (*n* = 3), BCA (*n* = 2) and CAB (*n* = 4), determining the order of the three intervention diets. In randomisation sequence ABC, one patient dropped out after completing the first intervention diet due to bad tolerance to the low polyol diet. In the same group, one patient unexpectedly changed randomisation sequence from ABC to BCA after completing the first intervention diet (Figure 2). No patients had comorbidities that could compromise the evaluation.

**Figure 3:**
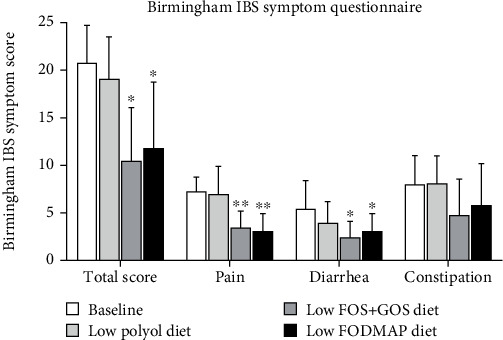
IBS symptom severity assessed by the Birmingham IBS questionnaire at baseline and after each intervention diet. Lower scores indicate improved IBS symptoms ⁣^∗^*p* < 0.05 and ⁣^∗∗^*p* < 0.01 as compared to baseline and analysed by the Wilcoxon signed-rank test for paired data. Total score and scores for three underlying variables (pain, diarrhoea and constipation) are presented as mean ± SD. IBS, irritable bowel syndrome; FOS, fructooligosaccharides; GOS, galactooligosaccharides.

Changes in IBS symptom severity assessed by the Birmingham IBS symptom questionnaire are shown in Figure 3. Compared with baseline (score: 20.8 ± 3.9), patients demonstrated a significant reduction in gastrointestinal symptoms on the low FOS + GOS diet and the low FODMAP diet (score: 10.5 ± 5.6 and 11.8 ± 7.0, respectively, *p* < 0.05). Further, the three underlying variables of the Birmingham IBS questionnaire (pain, diarrhoea and constipation) were also reduced compared to baseline on the low FOS + GOS diet (scor: pain, 7.3 ± 1.5 vs. 3.4 ± 1.8, *p* < 0.01; diarrhoea, 5.4 ± 3.0 vs. 2.4 ± 1.7, *p* < 0.01; constipation, 8.0 ± 3.0 vs. 4.8 ± 3.7, *p* > 0.05) and the low FODMAP diet (score: pain, 7.3 ± 1.5 vs. 3.0 ± 1.9, *p* < 0.05; diarrhoea, 5.4 ± 3.0 vs. 3.0 ± 1.9, *p* < 0.05; constipation, 8.0 ± 3.0 vs. 5.8 ± 4.4, *p* > 0.05); however, though constipation scores were numerically lower compared to both baseline and the low polyol diet (score: 8.1 ± 2.9), the differences were not statistically significant (Figure 3). No difference in Birmingham IBS symptom scores was found between the low FOS + GOS diet and the low FODMAP diet. Patients demonstrated significantly higher IBS symptom severity on the low polyol diet compared to both the low FOS + GOS diet and the low FODMAP diet (*p* < 0.05 and *p* < 0.05, respectively), and no change in Birmingham IBS symptom scores was found on the low polyol diet compared to baseline (score: 19.1 ± 4.4 vs. 20.8 ± 3.9).

When evaluating adequate relief (IBS-AR), none of the patients (0%) on the low polyol diet experienced adequate or satisfactory relief of their IBS symptoms. In contrast, the proportion of patients reporting adequate relief was 75% on the low FOS + GOS diet and 62.5% on the low FODMAP diet, which were significantly higher compared to the low polyol diet (*p* < 0.01 and *p* < 0.01, respectively). No significant difference in adequate relief was found between the low FOS + GOS diet and the low FODMAP diet.

Quality of life was significantly improved on the low FOS + GOS diet compared to baseline, with an overall IBS-QOL score of 25.5 ± 15.1 compared to 44.0 ± 13.2 at baseline (*p* < 0.05). The following subscale domains were also improved on the low FOS + GOS diet: body image (*p* < 0.05), health worry (*p* < 0.05) and sexual function (*p* < 0.01). The overall IBS-QOL score was numerically lower on the low FODMAP diet compared to baseline, but the difference was not statistically significant (score: 30.2 ± 19.6 vs. 44.0 ± 13.2). However, evaluating IBS-QOL subscale domains for the low FODMAP diet, body image and health worry improved statistically significantly compared to baseline (*p* < 0.05 and *p* < 0.05). No change in IBS-QOL scores was observed on the low polyol diet compared to baseline (score: 41.4 ± 11.2 vs. 44.0 ± 13.2) (Table 2).

## 4. Discussion

In this crossover study, we investigated if all carbohydrate groups eliminated in the low FODMAP diet have the same impact in relieving gastrointestinal symptoms in patients with IBS. Our main findings were that only eliminating oligosaccharides from the diet improved gastrointestinal symptoms equally to the restricted low FODMAP diet in patients with IBS. In addition, the proportion of patients who were satisfied with their symptoms was 75% on the low FOS + GOS diet and 62.5% on the low FODMAP diet, similar to prior studies assessing the effect of the low FODMAP diet in patients with IBS [15, 25, 26]. Results from the Birmingham IBS symptom questionnaire also indicated that the low FOS + GOS diet and the low FODMAP diet induce a similar reduction of gastrointestinal symptoms in patients with IBS, strengthening the conclusion. However, there is a lack of evidence on the general effect of polyols on intestinal function and conflicting results pertaining to polyol malabsorption in IBS [27]. Polyol malabsorption has been found to increase when polyols are ingested in combination with other carbohydrates and may induce laxative effects, as well as flatulence, bloating and abdominal discomfort in sensitive individuals [27, 28]. In addition, polyol ingestion can lead to intestinal dysmotility in patients with IBS [27]. However, when polyols were eliminated from the present study's diet, there was no improvement in any of the common symptoms of intestinal dysmotility (abdominal pain, nausea, vomiting, bloating and constipation). We found that solely eliminating polyols from the diet did not affect gastrointestinal relief of IBS symptoms or quality of life, indicating that polyols alone may not play a major role in the dietary management of IBS. Furthermore, a generalised alleviation of the low FODMAP diet was shown to reduce the beneficial effects of the treatment [29]. This adds to the conclusion that moderation of the low FODMAP diet should be conducted by allowing the intake of polyols in these highly selected patients referred for dietary changes. This finding is not in agreement with a study reintroducing sorbitol and finding abdominal pain within 1 day [18]. Probably, the dose of polyols is a determining factor.

Our findings point towards a specific combination of carbohydrates (FOS and GOS) as the main drivers responsible for provoking exacerbations of gastrointestinal symptoms in IBS. This is in line with a study by Biesiekierski et al. [30], who also suggest that oligosaccharides may be responsible for the symptoms experienced by patients with IBS [31]. This challenges the current FODMAP hypothesis, suggesting that IBS symptoms are induced in a dose-dependent manner and not by individual carbohydrates [32].

Although this study shows great potential to modify the low FODMAP and alleviate the extent of foods eliminated from the diet, there are some limitations to this study. Despite the crossover design of the study reducing the biological variation and the highly selected patients, the number of participants is low. However, a small sample showing clear results is not an indication of a Type 1 error. A disadvantage of the crossover design is the risk of carry-over effects between the three diets. This risk may be reduced by a washout period. However, it would be problematic, as the washout diet should be the habitual diet, and the patients were referred for help in changing that diet and paid for that. It is reasonable to assume that 4 weeks is sufficient to eliminate or markedly reduce a potential carry-over effect. In the study, significant differences in gastrointestinal symptoms are found between the low polyol diet and both the low FOS + GOS diet and low FODMAP diet, meaning that this carry-over effect is probably small. The study's external validity may be reduced by the fact that most participants were female, but that is the general finding in every investigation and very characteristic for Danish IBS patients. Furthermore, the recruitment model involved private clinics and some payment in a dominating free-of-charge public health system, which could give rise to selection bias with restricted possibilities to extrapolate the results to the total population of patients with IBS. The adherence to the diets with low GOS and FOS, however, was extremely good, probably due to a very high degree of motivation if treatment is paid for. However, despite this, the results of this study are clear and of a clinically relevant size of order despite the limited number of patients. The low polyol diet was related to poorer adherence than the others, also indicating that the patients did not find it useful. Nine patients were insufficient to define risk factors for different outcomes and for comparisons of subgroups.

## 5. Conclusion

Low FOS/low GOS diets improved gastrointestinal symptoms equally to the standard low FODMAP diet in patients with IBS, but polyol restriction did not have any clinically relevant effect. We recommend that the starting point in patients with IBS should be a low FOS/low GOS diet supplemented by standard dietary advice to IBS patients.

## Figures and Tables

**Table 1:** Baseline characteristics for the IBS patients testing components of the low FODMAP diet.

**Characteristics**	
Age (years)	38 ± 13
Gender (M/F)	(1/8)
BMI (kg/m^2^)	22.6 ± 1.8
Birmingham IBS symptom score	
Total score	20.8 ± 3.9
Pain	7.3 ± 1.5
Diarrhoea	5.4 ± 3.0
Constipation	8.0 ± 3.0
IBS-QOL, total score	44.0 ± 13.2

*Note: *Mean ± SD, *n* = 9.

Abbreviations: BMI, body mass index; IBS, irritable bowel syndrome; IBS-QOL, irritable bowel syndrome quality of life.

**Table 2:** Nine patients with IBS testing three variants of the low FODMAP diet in a crossover design. Health-related quality of life was assessed by the irritable bowel syndrome quality of life (IBS-QOL) questionnaire at baseline and after each intervention diet. IBS-QOL and eight domain scores. Lower scores indicate improved quality of life.

**Score**	**Baseline**	**Low polyol diet**	**Low FOS + GOS diet**	**Low FODMAP diet**
IBS-QOL	44.0 ± 13.2	41.4 ± 11.2	25.5 ± 15.1^∗^	30.2 ± 19.6
Dysphoria	41.3 ± 19.3	39.6 ± 17.7	24.2 ± 19.7	26.2 ± 23.3
Interference with activity	31.0 ± 19.0	30.6 ± 11.3	16.1 ± 12.7	24.1 ± 19.9
Body image	63.2 ± 17.8	58.3 ± 19.8	35.9 ± 25.4^∗^	35.2 ± 25.9^∗^
Health worry	57.4 ± 14.1	50.9 ± 24.5	31.2 ± 25.9^∗^	35.4 ± 24.3^∗^
Food avoidance	62.0 ± 18.7	61.1 ± 20.0	35.0 ± 24.1	50.0 ± 23.1
Social reaction	38.2 ± 20.6	34.0 ± 20.3	28.1 ± 23.4	28.1 ± 28.3
Sexual	40.3 ± 27.1	43.1 ± 15.5	17.2 ± 11.5^∗∗^	32.8 ± 35.9
Relationship	33.3 ± 24.6	27.8 ± 16.7	18.7 ± 19.8	24.0 ± 13.7

*Note: *⁣^∗^*p* < 0.05 and ⁣^∗∗^*p* < 0.01 compared to baseline and analysed by the Wilcoxon signed-rank test for paired data. IBS-QOL scores are presented as mean ± SD.

Abbreviations: FOS, fructooligosaccharides; GOS, galactooligosaccharides; IBS, irritable bowel syndrome.

## Data Availability

Data are available upon request from the authors.
